# Validation of markerless human pose estimation methods for clinical assessment of elbow range of motion

**DOI:** 10.1371/journal.pone.0353801

**Published:** 2026-07-23

**Authors:** David Rode, Romina Willi, Eva Schulz, Tim Schneller, Philipp Moroder, Laurent Audigé, Mario Bizzini, Nicola A. Maffiuletti, Peter Wolf, Robert Riener

**Affiliations:** 1 Sensory-Motor Systems Lab, ETH Zurich, Zurich, Switzerland; 2 Human Performance Lab, Schulthess Klinik, Zurich, Switzerland; 3 Kardinal Schwarzenberg Klinikum, Schwarzach im Pongau, Austria; 4 Research and Development, Schulthess Klinik, Zurich, Switzerland; 5 Surgical Outcome Research Center, University Hospital of Basel, Basel, Switzerland; 6 Balgrist University Hospital, University of Zurich, Zurich, Switzerland; New Jersey Institute of Technology, UNITED STATES OF AMERICA

## Abstract

Clinicians rely on the assessment of joint range of motion to determine the necessity of clinical interventions and to track rehabilitation progress. They often perform visual assessments of these joint angles due to limited time for longer but more accurate methods. Markerless monocular human pose estimation methods could help reduce the workload of physicians in clinical practice, allow patients to conduct regular assessments remotely, and be used to provide reliable assessments in the absence of expert raters, but they have not been validated extensively on clinical populations. We measured the elbow flexion and extension ranges of motion of 46 patients with shoulder and elbow pathologies. These values were assessed by RTMW and HSMR, two different monocular pose estimation methods, and an experienced physician who performed visual assessments. A state-of-the-art passive-marker optical motion capture system was used as the reference system. For elbow flexion, HSMR outperformed visual assessment. HSMR had a higher concordance correlation coefficient of 0.92 and a lower minimal detectable change of 6.74° as determined by a concordance analysis and a linear mixed-effects model. Conversely, for elbow extension, visual assessment outperformed both markerless methods. RTMW was the best-performing markerless method with a concordance correlation coefficient of 0.83 and a minimal detectable change of 7.93°. Thus, HSMR represented a viable alternative to visual assessment for measuring elbow flexion range of motion in a clinical population, but neither RTMW nor HSMR offered an improvement over visual assessment for measuring elbow extension range of motion.

## Introduction

### Assessments of range of motion

The range of motion (ROM) of joints is one of the most frequently measured functional metrics [1,2] and plays an important role in clinical practice and biomechanical research. Accurate ROM measurements are important for the correct diagnosis of impairments [3,4], determining the outcome of clinical interventions [3] and patient satisfaction [5], and tracking the progress of rehabilitation [6]. In general, ROM can be measured in two different ways: if participants move as far as possible without outside help, it is known as active ROM. If outside support is provided to the participant, the measurement is referred to as passive ROM [3]. This study investigated the measurement of active elbow flexion and extension ROM in patients. The clinical elbow flexion ROM used in this study is defined as the angle between the neutral position, corresponding to the forearm being aligned with the upper arm and stretched out in the direction of gravity while standing upright (see S1 Fig, a), and the point of maximal elbow flexion (see S1 Fig, b). Accordingly, clinical elbow extension ROM is defined as the angle between the neutral position and the point of maximal elbow extension. If the participant could not reach the neutral position, the clinical elbow ROM was registered as negative (see S1 Fig, c).

Due to their low cost and ease of use, universal goniometers are commonly used in clinical practice [7]. Their intra-rater reliability has been shown to be high when measurements are performed by the same rater [7,8]. However, they require the use of both hands, which limits the support clinicians can provide to the patient, and clinicians must visually estimate the center of rotation of joints and visually align the goniometer arms with the limbs of the patient, which limits their accuracy and repeatability [1]. Furthermore, goniometers suffer from low inter-rater reliability due to differences in how clinicians position patients, how they locate anatomical landmarks on patients, and how they are affected by cognitive bias [7,8]. This low inter-rater reliability necessitates having a single rater perform all measurements when using a goniometer to assess the effect of a treatment on a patient [9]. Goniometers have been validated using radiographic measurements, revealing a mean difference of −2.4° when measuring elbow flexion ROM with limits of agreement between −9.5° and 4.6° [8]. The elbow extension ROM was measured with a mean difference of −1.3° and limits of agreement between −11.4° and 9.2°.

Clinicians frequently perform visual assessments due to their short duration, ease, and lack of necessary measurement equipment [10]. As visual assessment is the most commonly used method in surgical settings, it can be considered the standard of care (SOC) [10]. A prior study investigated the accuracy of visual assessments and goniometers for elbow flexion and extension ROM in cadavers with a passive-marker optical motion capture system (OMC) as ground truth [10]. Visual assessments by multiple raters of different levels of experience showed a mean absolute difference of 8.4° in elbow flexion ROM and a mean absolute difference of 12.2° in elbow extension ROM compared to OMC. These differences were slightly higher than the measurements with a goniometer, which showed a mean absolute difference of 7.4° in elbow flexion and 10.5° in elbow extension. The authors found visual assessments to be clinically equivalent to goniometers in accuracy. Another study found that the accuracy of visual assessments performed by experienced physicians was equal to the accuracy when using handheld short-arm goniometers [11]. They found that visual and goniometer measurements both performed by the same experienced surgeon showed a mean difference of 1° for elbow flexion ROM with limits of agreement between −6° and 7°. When measuring elbow extension ROM, the mean difference between both methods was found to be 1° with limits of agreement between −7° and 9°. Goniometry and visual estimation were shown to have low agreement between raters with different levels of experience [11] and both can be affected by cognitive bias [12]. Blonna et al. suggested that visual estimation showed systematic errors when raters lacked training and experience and recommended that only a single rater should perform all ROM measurements of a patient [11].

Several studies have assessed the accuracy of photography and teleconference goniometry, wherein a clinician measures the joint ROM on captured still images or during a live video transmission, respectively. Dent et al. conducted a study with 48 healthy participants and found that videoconferencing performed similarly to photography. Videoconferencing showed a correlation coefficient of 0.93 for elbow flexion and 0.86 for elbow extension when using clinical goniometry as a reference, while photography had a correlation coefficient of 0.73 for elbow flexion and 0.82 for elbow extension [13]. Note that this study used goniometers placed on digital images to measure ROMs. Meislin et al. investigated the accuracy of photography when using a digital tool to measure elbow ROM and compared it with conventional goniometry in 32 healthy participants. They found a mean difference of 3°–3.5° between photography and goniometry with an intraclass correlation coefficient between 0.828 and 0.740 and a correlation coefficient between 0.845 and 0.757 [14].

### Human pose estimation

The two most common measurement methods for ROM, visual assessment and goniometry, require a clinician to be present to take measurements. Novel markerless measurement methods, such as human pose estimation (HPE), could provide ROM measurements without the need for clinicians to perform them, thereby reducing their workload and enabling remote and more continuous measurements than currently possible. As these methods are deterministic models and thus repeatable, they could lead to an increase in intra-rater reliability. Furthermore, these models only use visual inputs and are not aware of the clinical context of the patient, which reduces the influence of cognitive bias on measurements. When using the same HPE method for all measurements on the same patient, the advised use of a single rater could easily be achieved even in clinical practice, thereby potentially eliminating issues with inter-rater reliability.

HPE methods have increasingly improved in accuracy with the adoption of new model architectures and novel approaches to landmark detection. The field of HPE includes both skeleton-based and mesh-based approaches [15]. Whereas skeleton-based methods aim to determine human landmarks within a given input image, mesh-based methods determine the shape of a visible human body using parametric body models [15]. Most modern HPE methods rely on convolutional neural networks to extract image features from visual inputs. These features are then used to either classify landmarks, directly regress landmarks, or generate probability heatmaps of landmarks. Skeleton-based methods return the location of joint centers in pixels or metric units, whereas mesh-based methods return a surface mesh of the detected human body.

Several studies have assessed the accuracy of markerless methods at determining elbow ROM. Wang et al. determined the accuracy of Blazepose, a markerless monocular HPE method, when measuring joint ROM in 25 healthy participants using passive-marker OMC as a reference [16]. They found an inter-rater reliability of 0.53 for elbow flexion ROM using the two-way mixed-effects intraclass correlation coefficient. The standard error of measurement by Blazepose was measured as 11.69° and the minimal detectable change was found to be 32.40°. Kim et al. determined the optimal viewing angle when using Blazepose to measure joint angles by comparing it to passive-marker OMC on 10 healthy participants [17]. This study found that the viewpoint on the sagittal plane was the most accurate when measuring elbow flexion, with a mean absolute difference of 23.18° and a correlation coefficient of 0.88. It should be noted that this study evaluated the accuracy for sequences of joint angles over time and not for singular ROM values. Ryu et al. used MMPose as the monocular HPE method and compared shoulder and elbow ROM measurements with conventional goniometry by expert raters. On a dataset of 25 patients, they found an intraclass correlation coefficient of 0.996 for elbow flexion and 0.985 for elbow extension [18]. Lim used OpenPose as the HPE method on multi-view images to measure the elbow angle of a healthy participant. Using passive-marker OMC as a reference, they found no statistically significant differences in the measured maximal and minimal elbow angles nor in the elbow ROM [19].

So far, monocular HPE methods have not been validated for measuring elbow flexion and extension ROM against an OMC reference system on a clinical population. This study therefore determined the accuracy, precision, and concordance of HPE methods on a clinical population using a passive-marker OMC system as the reference system, and compared them to visual assessment by an expert physician, representing the current standard of care. HPE methods could provide a viable alternative to clinical ROM measurements if their performance matches that of visual assessment.

## Methods

### Ethical statement

This study was approved by the Ethics Committee of the Canton of Zurich (BASEC 2023−01448) and was carried out following all relevant guidelines and the Declaration of Helsinki. All participants provided their informed written consent to participate in this study.

### Dataset

We recruited 46 patients from the Schulthess Klinik in Zurich to participate in this study (see Table 1) from the 12th of October 2023 to the 21st of December 2023. These patients were attending surgical consultations at the time of measurement. The inclusion criteria were an age of at least 18 years, sufficient communication in German, and a unilateral orthopedic upper limb pathology (e.g., rotator cuff lesion, instability, osteoarthritis). Patients were excluded from this study if they suffered from bilateral pathologies or were advised to abstain from active movements by their treating physician. A subset of 36 patients was being treated for a shoulder pathology, while 10 patients were being treated for an elbow pathology. We included patients with shoulder pathologies as there is a correlation between reduced shoulder function and elbow injuries [20] and to increase the generalizability of our results to a wider clinical population.

**Table 1:** Characteristics of the participants.

Characteristic	Mean [range]
Age (years)	52.1 [20.9–82.7]
Height (cm)	175 [150–210]
Weight (kg)	80 [40–125]
Body mass index (kg/m^2^)	26.1 [17.3–39.1]
**Characteristic**	**Number (percentage)**
Sex	16 female (35%), 30 male (65%)
Dominant hand	3 left (7%), 43 right (93%)
Affected side	18 left (39%), 28 right (61%)
Dominant side affected	25 yes (54%), 21 no (46%)

Overview of the mean and range of study participant characteristics. In total, 46 patients in treatment for unilateral elbow or shoulder pathologies were included in this study.

### Data collection and processing

**Fig 1:**
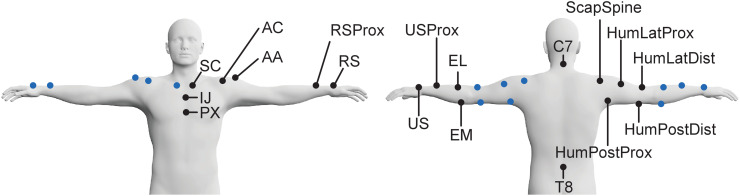
Marker setup for optical motion capture. Passive markers used for optical motion capture: location and names of the 32 markers of the University of Southampton Upper Limb Kinematic Model [21]. Markers were attached to the skin of participants. The blue markers are symmetric counterparts to the black, annotated markers.

Each of the participants was measured in a single session in the presence of a surgeon with six years of experience in orthopedic and trauma surgery. The participants were instructed to perform elbow flexion and elbow extension starting from a neutral position with their arms at their sides while standing (see S1 Fig, a). All motions were performed bilaterally to limit compensatory movements, but only the ROM of the affected side was measured. After giving the participants a command, they moved both arms from the neutral position to the maximal possible flexed or extended position without feeling pain (see S1 Fig, b and c). The participants were free to choose their preferred forearm orientation during the movements. After holding the maximal position for a short duration, they returned to the neutral position and rested. The maximal active ROM was recorded by three different methods simultaneously: a passive-marker OMC system, an RGB camera, and the participating surgeon performing a visual assessment. Each measurement was repeated three times whenever possible under identical settings. In case participants were not able to perform all repetitions, the number of repetitions was reduced. The selected statistical methods required repeated measurements, but they did not require an equal number of repetitions for all participants, so no data points were removed in case a participant could not complete all repetitions. Passive-marker OMC is widely used in clinical and research settings for assessing joint kinematics and was commonly used in related studies as a reference system, which is why we selected it as the reference system for this study [10,16,17]. Our OMC system (Vicon, Oxford, United Kingdom) consisted of 15 infrared cameras running at a sampling frequency of 200 Hz. We used the University of Southampton Upper Limb Kinematic Model (distributed by Vicon, Oxford, United Kingdom and developed by Warner et al. [21]), which requires a set of 32 reflective markers on the participants (see Fig 1). This kinematic model is based on the International Society of Biomechanics (ISB) standards for joint angle definitions [22] and defines the sequence of the rotations in the elbow joint in the order of first flexion/extension, then pronation/supination, and lastly a passive carrying angle. Participants were topless or wore minimal clothing and markers were attached directly to the skin whenever possible. After labeling the markers, the joint angles were extracted from the kinematic model (Vicon Nexus 2.12, Oxford, United Kingdom). As these joint angles were defined according to ISB standards [22], we converted them to the clinical definition of the elbow ROM, which we used consistently throughout this study (see S1 Fig). Following this clinical definition, elbow flexion ROM was measured positively from the neutral position to the maximally flexed arm position. The elbow extension ROM was measured positively starting from the neutral position towards the maximally hyperextended arm position. Therefore, negative elbow extension ROM values refer to the situation when participants were not able to extend their elbow beyond the neutral position.

The RGB camera (Logitech C920 HD Pro, Lausanne, Switzerland) was placed at a height of 0.9 m and was facing the sagittal plane on the affected side of each participant at a distance of 3 m. In a previous study, we found this camera orientation to be the most accurate when measuring elbow flexion and extension [23]. The videos were captured at a frame rate of 30 FPS (OBS Studio, OBS Studio Contributors, Open Source).

The surgeon selected their preferred viewpoint and observed the motions of the participants without obstructing the camera. After observing the motions, the surgeon estimated the elbow flexion ROM in steps of 5° and the elbow extension ROM in steps of 1°. We requested the surgeon to measure elbow flexion ROM in steps of 5° based on the recommendations of Blonna et al., who concluded that this approach simplified recordings, comparisons, communication, and recollection without a loss of accuracy [11]. In their study, the rater with the highest accuracy and best intra-rater reliability estimated elbow flexion ROM in steps of 5°. As the ROM of elbow extension is smaller than that of elbow flexion, we requested the surgeon to measure elbow extension ROM in steps of 1°.

The video recordings from the RGB camera were used as input for the selected monocular HPE methods. In this study, we investigated two monocular HPE methods: Real-Time Multi-person Whole-body Pose Estimation models (RTMW) and Human Skeleton and Mesh Recovery (HSMR).

We selected RTMW [24] using its ‘performance’ setting based on a prior benchmarking study in which it demonstrated higher accuracy relative to other state-of-the-art HPE methods on typical physiotherapeutic movements [25]. RTMW is a skeleton-based HPE method, which returns 134 two-dimensional landmarks in pixel coordinates. The elbow angle on the affected side was determined using the scalar product of vectors connecting the wrist, elbow, and shoulder. This angle was then converted into the respective ROM value based on the clinical definition of elbow extension and flexion ROM. We used the default settings of RTMW and did not perform any additional training or fine-tuning of the model. The strength of RTMW is its high inference speed while retaining high accuracy, which makes it suitable for real-time applications. However, it does not fit a kinematic model to the detected landmarks, which can lead to nonphysiological joint positions and angles.

Therefore, we decided to include an HPE method with a kinematic body model in this study, which can provide more biomechanically plausible joint angles. We chose HSMR [26] because it uses the “Skeletal Kinematics Enveloped by a Learned body model” (SKEL) [27] human model, which offers vast improvements over the commonly used “Skinned Multi-Person Linear Model” (SMPL) human model, due to its biomechanical joint angle definitions. SMPL is a widely used parametric body model that represents the human body surface as a mesh with 23 joints using 72 pose parameters [28]. The use of the SMPL model for biomechanical applications is limited due to its joint definitions, which do not correspond to anatomical joint locations and because it models all joints as ball-and-socket joints [27]. SKEL is based on SMPL but enhances it by defining 24 anatomically accurate joints, resulting in a reduced set of 46 pose parameters, and modeling them with appropriate kinematic constraints, such as the knee being a hinge joint [27]. HSMR is a mesh-based HPE method, which reconstructs a human mesh from a monocular image and fits SKEL to the mesh. We extracted the elbow joint angles directly from the outputs of the biomechanical model and then converted them to the clinical ROM definitions for flexion and extension ROM. As with RTMW, we used the default settings of HSMR and did not perform any additional training or fine-tuning of the model. The strength of HSMR is its fit of a biomechanical model to the detected human body, which can lead to more accurate and physiologically plausible joint angles. However, the mesh estimation process and the subsequent fitting of the kinematic model are computationally intensive and time-consuming, which limits its use for real-time applications.

### Statistical analysis

First, we created boxplots of the absolute ROM values of all repetitions measured by OMC, RTMW, HSMR, and SOC to visualize the distributions of measured ROM values. By inspecting the raw data, we identified three flipped sign errors, which all occurred during the visual estimation of elbow extension ROM. These extension ROM entries were erroneously recorded as positive, which placed them far beyond the range of physiological hyperextension according to the clinical definition. Upon visual inspection of the original video recordings, we confirmed that the surgeon intended to enter these entries as negative, which corresponded to participants not reaching the neutral position when extending their elbows. We corrected these three entries by flipping their signs to negative and keeping their absolute values unchanged. In total, three entries out of 131 were corrected due to flipped signs, representing 2.3% of all elbow extension ROM measurements by SOC. No other data cleaning or outlier removal was performed for the SOC, OMC, RTMW, or HSMR measurements.

We computed the mean difference, median difference, mean absolute difference, and root mean square difference of all investigated methods to provide simple grand mean comparisons because these metrics are commonly reported.

As all measurements were repeated multiple times, a conventional Bland-Altman analysis would not capture the underlying structure of the data [29]. We decided to model the data using linear mixed-effects models as is recommended for repeated measurements [30]. We assumed that the ROM measurements for both motions, namely elbow flexion ROM and elbow extension ROM, for participant *i* and measurement method *j* at time *t* could be modeled as:


$$\begin{array}{c} {ROM}_{ijt}^{motion}=\mu +\alpha_{i}+\beta_{j}+(\alpha \beta {)}_{ij}+\varepsilon_{ijt} \end{array}$$ (1)

Here, μ represents the adjusted mean value of the reference OMC method for the respective motion, $\alpha_{i}$ is the random effect of the participants, $\beta_{j}$ is the fixed effect of the respective measurement method representing the systematic constant bias of the method compared to OMC, $(\alpha \beta {)}_{ij}$ is the random interaction between participants and measurement methods, and $\varepsilon_{ijt}$ is the residual error. We assumed that the participants represented a sample from a larger relevant patient population and, therefore, we considered them as a random effect. The investigated measurement methods were the only methods of interest in this study and did not represent samples from a larger population of measurement methods. Therefore, we considered the three investigated measurement methods to be fixed effects. Since we performed all repeated measurements during the same session with sufficient rest for the participants between repetitions, we assumed that there was no effect of time on the measured ROM values. Instead, we treated the repeated measurements as identical replicates with an insignificant order of measurements and therefore did not include time as a fixed effect nor did we include random interactions involving time.

The concordance correlation coefficient (CCC) and its confidence interval were determined using the cccrm library (version 3.0.5, [31]) in R (version 4.5.1, The R Foundation for Statistical Computing, Open Source) for repeated non-longitudinal measurements and considering participant-method interactions, which used the same model as defined in Eq. 1. We fitted each measurement method separately from the other methods for each motion using OMC as the reference method. The confidence intervals of the CCC values were determined by non-parametric bootstrapping with 10,000 simulations and participant-wise resampling and adjusted using the bias-corrected and accelerated bootstrap interval method (BCa).

In order to determine the fixed effects $\beta_{j}$, their p-values, and additional accuracy and precision metrics, we implemented the derived linear mixed-effects model (see Eq. 1) using the lme4 library (version 1.1.37, [32]) in R (version 4.5.1, The R Foundation for Statistical Computing, Open Source) as:


$$\begin{array}{c} {ROM}_{ijt}^{motion}\sim method+(1|participant)+(1|method:participant) \end{array}$$

Each measurement method was fitted separately for elbow flexion and elbow extension and separate from the other methods using OMC as the reference. The p-values of the fixed effects were determined using the lmerTest library (version 3.1.3, [33]), which used a Satterthwaite approximation. The quality of the fit of the models was determined using the marginal and conditional *R*^2^ metrics using the MuMIn library (version 1.48.11, [34]). We extracted the fixed effects $\beta_{j}$, the standard deviations of the participant effect $\sigma_{\alpha }$, the standard deviations of the participant-method interaction $\sigma_{\alpha \beta }$, and the standard deviations of the residual error $\sigma_{\varepsilon }$ from the fitted models. From these, we computed the mean squared deviation (MSD), coverage probability (CP), total deviation index (TDI), and minimal detectable change (MDC) using the formulas derived by Parker et al. [30].

The CP can be calculated using the MSD and the range of clinically acceptable differences δ. In the following equations Φ is the standard normal cumulative distribution function. The minimal clinically important difference (MCID) was assumed to be 10° for elbow flexion ROM and 5° for elbow extension ROM following Russo et al. [10] and inserted as value for δ.

We thus computed the metrics using OMC as the reference method as follows:


$$\begin{array}{c} MSD(OMC,{method}_{j})=(\beta_{OMC}-\beta_{j}{)}^{2}+2(\sigma_{\alpha \beta }^{2}+\sigma_{\varepsilon }^{2}) \end{array}$$ (2)

$$\begin{array}{c} CP(\delta )=1-2[1-\Phi (\frac{\delta }{\sqrt{MSD(OMC,{method}_{j})}})] \end{array}$$ (3)

The TDI can be computed from the MSD and a specified proportion $p_{TDI}$. We set $p_{TDI}$ to 0.95:


$$\begin{array}{c} TDI(p_{TDI})=\Phi^{-1}(\frac{1+p_{TDI}}{2})\cdot \sqrt{MSD(OMC,{method}_{j})} \end{array}$$ (4)

Furthermore, we computed the standard error of measurement (SEM) and the resulting 95% MDC using the formulas from Caronni et al. [35]:


$$\begin{array}{c} SEM=\sqrt{\sigma_{\varepsilon }^{2}} \end{array}$$

$$\begin{array}{c} 95\%\;MDC=1.96\cdot \sqrt{2}\cdot SEM \end{array}$$

We used a non-parametric bootstrap approach with 10,000 simulations and participant-wise resampling to determine the confidence intervals for all the previously mentioned metrics. Bootstrapping was implemented with the boot library (version 1.3.31, [36]) and adjusted using the BCa bootstrap interval method. All point estimates and bootstrap simulations were performed with the ‘bobyqa’ optimizer and 2e5 maximum iterations (see S2 Fig for the distributions of the bootstrapped metrics and their confidence intervals).

We checked the validity of the main assumptions of linear mixed-effects models, namely a linear relation between the ROM response and the explanatory variables, a constant variance of the response, and the error and random effect coefficients being distributed independently and normally [37]. These assumptions were verified using plots of the conditional raw residuals over the fitted ROM values and Q-Q plots of the conditional raw residuals and random effects (see S3 Fig). These plots were created with the redres library (version 0.0.0.9, [38]) in R (version 4.3.3, The R Foundation for Statistical Computing, Open Source). The conditional raw residuals in all models were centered around zero, which supported the assumption of linear relations. Furthermore, no obvious non-uniform distributions were visible from the conditional raw residuals, which supported the assumption of constant variance. The Q-Q plots revealed that the residuals were primarily within the 95% confidence bands of a normal distribution for a wide range of values. Note that the SOC measurements showed a larger deviation from the normal distribution than the other measurement methods. Nevertheless, we opted for linear mixed-effects models without performing any data transformations as studies revealed that these models are largely robust to non-normal error distributions [39,40]. Schielzeth et al. recommended avoiding data transformations as these reduce the interpretability of results and because violations of the assumption of normally distributed errors are not impactful besides resulting in a greater variability of results [39]. Knief et al. recommended using linear mixed-effects models with normal distributions even when the assumptions for these models are violated. They argue that alternative models with other distribution assumptions or more complex fitting techniques might be more error-prone with less obvious consequences [40]. The Q-Q plots of the random effects showed good adherence to the assumption of normal distribution as well, as most were within the 95% confidence bands. Some large outliers were visible in the lower range of values, which could be explained by the relatively sparse data we collected from small ROMs.

In addition to the linear mixed-effects models, we performed a Taffé analysis [29], which requires repeated measurements and is capable of determining non-constant bias and non-constant precision. The Taffé method decomposes the total bias into a differential bias, which is a constant offset across the full measurement range, and a proportional bias, which is a component that scales linearly with the true ROM value as follows:


$$\begin{array}{c} bias=differential\;bias+(proportional\;bias-1)\cdot true\;ROM \end{array}$$ (5)

Using this method, we visualized and quantified the bias and precision of the different measurement methods compared to the reference OMC method. Note that the Taffé method determines the bias and precision with respect to true values and not the reference measurement values. In our case this means that the Taffé method determines the bias and precision of the investigated methods with respect to the true elbow flexion and extension ROM values and not the values measured by OMC. The Taffé method estimates the true values by computing the best linear unbiased prediction using only the reference OMC measurements. The analysis was performed using the MethodCompare library (version 1.1.0, [41]) in R (version 4.5.1, The R Foundation for Statistical Computing, Open Source).

## Results

**Fig 2:**
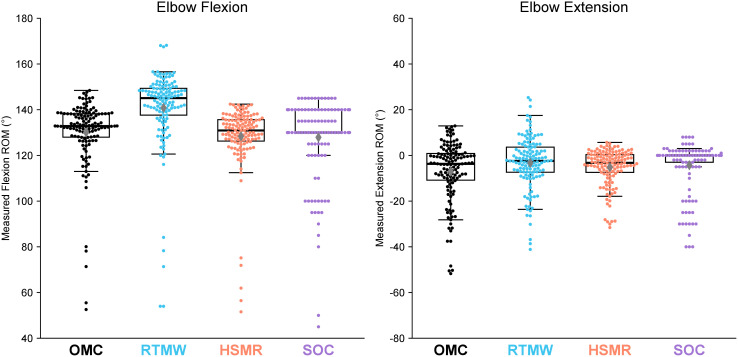
Measured elbow flexion and elbow extension ROM. Boxplots of ROM measured by passive-marker optical motion capture, monocular human pose estimators RTMW and HSMR, and the current standard of care of visual assessment. ROM was defined as depicted in S1 Fig. The boxes reach from the first to the third quartile, the solid lines indicate the median, the gray diamonds represent the mean, and whiskers include values within 1.5 times the interquartile range.

The mean value of elbow flexion ROM measured by OMC averaged over all repetitions and all participants was 130.05° and the median was 132.83°. For elbow extension, the mean value of all repetitions over all participants measured by OMC was −7.03° and the median value was −3.70° (see Fig 2).

Due to fatigue or pain, seven participants were only able to repeat the elbow flexion and elbow extension motions twice instead of the three times requested.

**Fig 3:**
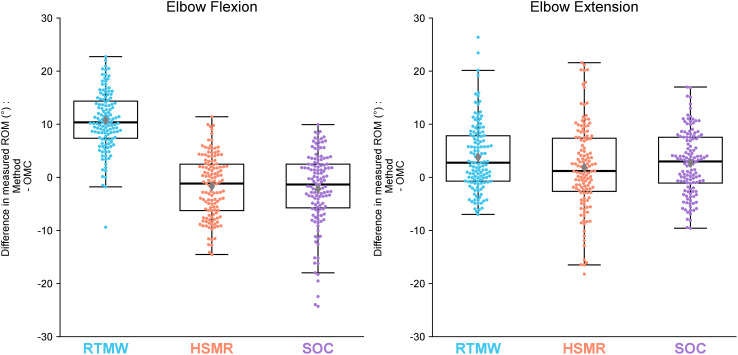
Differences in measured ROM. Boxplots of the measurement differences between the investigated measurement methods and the reference OMC measurement. The boxes reach from the first to the third quartile, the solid lines indicate the median, the gray diamonds represent the mean, and whiskers include values within 1.5 times the interquartile range.

When assessing the differences of each method compared to the standard OMC method, it is apparent that they differed in distribution, as well as in their means and medians (see Fig 3). No obvious large outliers remained after the sign errors were cleaned up.

Using the determined measurement differences, we report the mean difference, median difference, mean absolute difference, and root mean square difference of each method for both motions. These accuracy metrics allow simple first comparisons using the entire dataset without considering the structure of the data with respect to repeated measurements (see Table 2). HSMR showed the highest accuracy in elbow flexion for all computed metrics. For elbow extension, HSMR showed the best mean and median difference, but RTMW and notably SOC outperformed it in mean absolute difference and root mean square difference. SOC had the best mean absolute difference and root mean square difference for elbow extension.

**Table 2:** Accuracy metrics of the investigated methods using OMC as the reference method. The best value for each motion is underlined.

		RTMW	HSMR	SOC
Elbowflexion ROM	Mean difference (°)	10.75	−1.70	−2.15
	Median difference (°)	10.35	−1.17	−1.35
	Mean absolute difference (°)	10.94	4.95	5.42
	Root mean square difference (°)	12.18	6.01	7.31
Elbow extension ROM	Mean difference (°)	3.78	1.86	2.72
	Median difference (°)	2.75	1.19	2.99
	Mean absolute difference (°)	5.47	6.31	5.20
	Root mean square difference (°)	7.38	8.28	6.47

The linear mixed-effects models revealed HSMR to have the smallest fixed effect size for both motions (see Table 3). A low fixed effect size indicates a small bias compared to OMC, with the notable assumption of bias being constant across the entire measurement range. All models showed a high quality of fits with an $R_{conditional}^{2}$ of at least 0.951 for both motions. The magnitude of fixed effects, i.e., the constant bias of each method to the reference OMC method, was statistically significant for all investigated measurement methods and motions, except for elbow extension when measured by HSMR.

**Table 3:** Overview of fitted linear mixed-effects models. The table presents fixed effect magnitudes $\beta_{j}$ with their 95% bootstrap BCa confidence intervals (BCa CI), the standard errors (SE) of each estimated fixed effect, their t-values and p-values, the random effect standard deviations $\sigma_{\alpha }$ and $\sigma_{\alpha \beta }$, and the standard deviation of the residual error $\sigma_{\varepsilon }$. The smallest absolute fixed effect value for each motion is underlined, which indicates the method with the least constant bias to the reference OMC method when assuming the bias to be constant.

Motion	Method	$\beta_{j}$ (95% BCa CI) (°)	SE (°)	*t*-value	*p*-value	$\sigma_{\alpha }$ (°)	$\sigma_{\alpha \beta }$ (°)	$\sigma_{\varepsilon }$ (°)	$R_{marginal}^{2}$	$R_{conditional}^{2}$
Elbow flexion ROM	**RTMW**	10.69 (9.56, 11.89)	0.79	13.51	$1.75\times 10^{-17}$	17.24	3.49	2.48	0.083	0.982
	**HSMR**	−1.73 (−2.88, −0.47)	0.80	−2.16	$3.61\times 10^{-2}$	15.58	3.55	2.43	0.003	0.977
	**SOC**	−2.30 (−3.68, −0.78)	0.95	−2.41	$2.00\times 10^{-2}$	16.81	4.23	2.88	0.004	0.973
Elbow extension ROM	**RTMW**	3.91 (2.61, 5.16)	0.85	4.59	$3.62\times 10^{-5}$	11.88	3.71	2.86	0.023	0.951
	**HSMR**	2.09 (0.33, 3.86)	1.17	1.79	$8.10\times 10^{-2}$	9.60	5.44	2.29	0.009	0.959
	**SOC**	2.83 (1.59, 4.05)	0.82	3.44	$1.26\times 10^{-3}$	11.65	3.76	2.02	0.013	0.974

**Fig 4:**
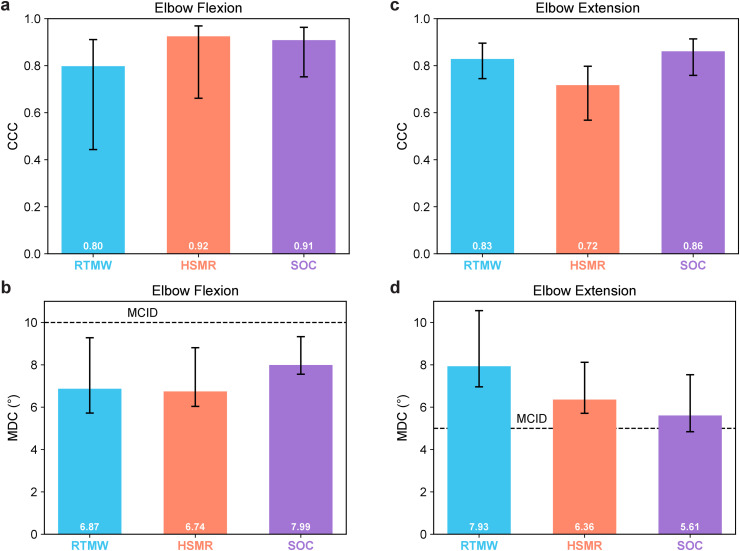
Concordance correlation coefficient and minimal detectable change. CCC and MDC values of the investigated methods with their corresponding bootstrapped 95% BCa confidence intervals. HSMR showed the highest CCC and smallest MDC for elbow flexion. Visual assessment had the highest CCC and lowest MDC for extension. Note that no method achieved the minimal clinically important difference for elbow extension ROM.

The CCC values revealed that HSMR had the highest concordance with OMC for elbow flexion with a value of 0.92, while SOC had the highest concordance for elbow extension with a value of 0.86 (see Fig 4 and S4 Table). HSMR had the smallest MDC for elbow flexion with 6.74°, while SOC had the smallest MDC for elbow extension with 5.61° (see Fig 4 and see S4 Table for the values of all computed metrics and their 95% BCa confidence intervals).

**Fig 5:**
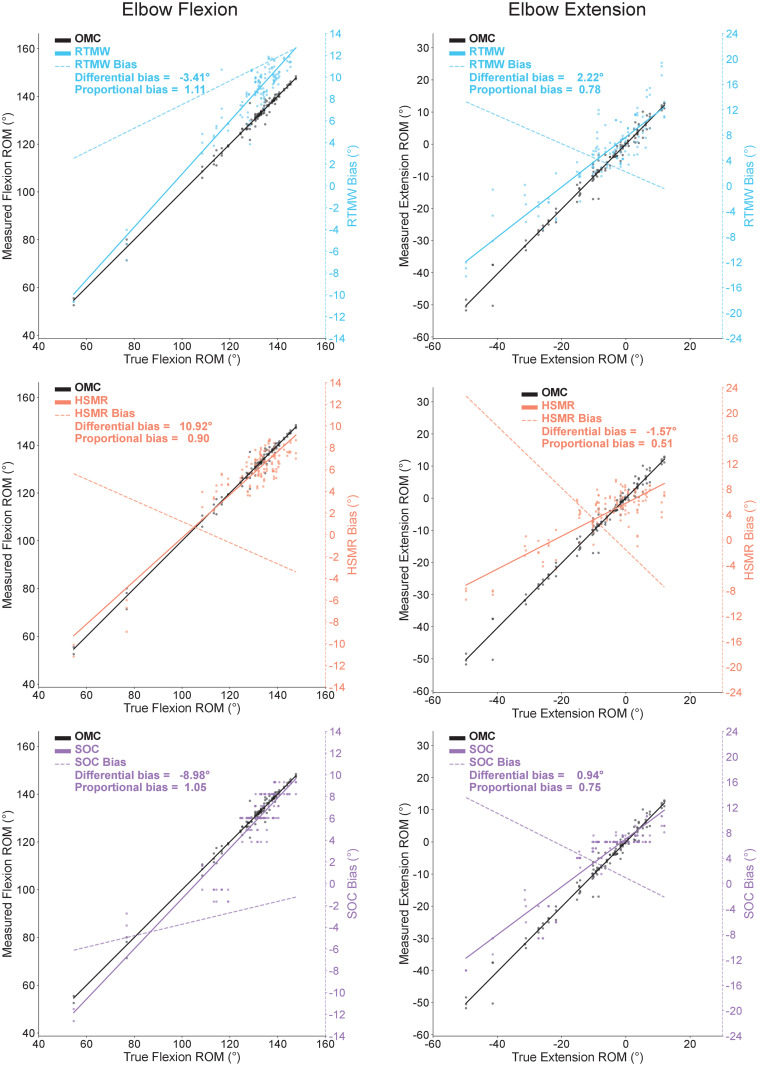
Bias from Taffé analysis. Non-constant bias of RTMW, HSMR, SOC, and OMC as determined by the Taffé analysis. The circles represent all measurements by the investigated methods against the true ROM values. The Taffé analysis estimates the true ROM values by computing the best linear unbiased prediction using only the reference OMC measurements. Solid lines are linear regressions of the measured ROM values against the true ROM values and are read against the left vertical axis. Dashed lines depict the bias between the investigated methods and the true values and are read against the right vertical axis. The Taffé analysis decomposes the bias into a differential bias, which is constant across the full measured ROM range, and a proportional bias, which scales linearly with the true ROM value (see Eq. 5).

The Taffé analysis revealed RTMW and SOC to both have an increasing bias when measuring elbow flexion, i.e., a proportional bias component larger than 1. For both methods, the bias increased with an increasing true flexion ROM (see Fig 5). RTMW consistently overestimated the true flexion ROM, and the amount of overestimation grew with increasing true flexion ROM, reaching approximately 13° at the largest flexion angles. SOC consistently underestimated the true flexion ROM, but its value of underestimation approached zero for increasing true flexion ROM. In contrast, HSMR showed a decreasing bias, i.e., a proportional bias smaller than 1, switching from overestimation to underestimation of the true flexion ROM at approximately 113°.

For elbow extension, all three measurement methods showed a decreasing bias with their proportional bias components all being smaller than 1 (see Fig 5). HSMR showed the largest proportional bias, substantially overestimating extension ROM smaller than −30° by more than 12° and underestimating extension ROM larger than 7° by more than 5°. SOC overestimated extension ROM smaller than approximately 4° and underestimated extension ROM larger than that. RTMW overestimated extension ROM smaller than approximately 10° and underestimated extension ROM larger than that.

**Fig 6:**
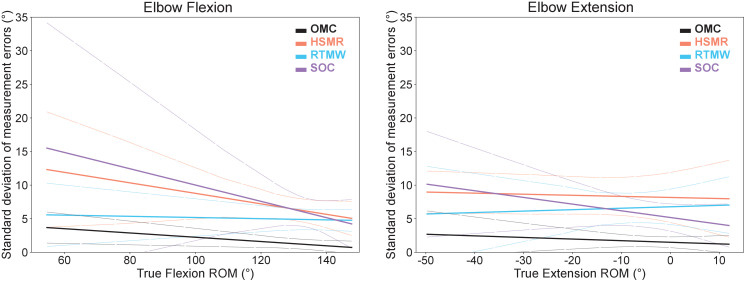
Precision from Taffé analysis. The solid lines represent the precision of each method as standard deviation of the respective measurement error over the range of true ROM values. The dashed lines indicate the 95% confidence interval of the respective precision curve. Black indicates the OMC reference method.

For elbow flexion, SOC showed the lowest precision across almost the entire range of measurements, though it improved with increasing true flexion angles (see Fig 6). RTMW showed the highest precision across most of the measured flexion ROM range, remaining nearly constant at around 5° to 5.5°. The precision of HSMR was higher than that of SOC for true flexion angles below 129°, with SOC being slightly more precise for true flexion angles larger than 129°. For elbow extension, RTMW showed the highest precision of all investigated methods for true extension angles below −13°, while SOC was the most precise above this threshold. HSMR showed the lowest precision across nearly the entire range of measured extension ROM. Note that the Taffé analysis also determined the precision of the reference OMC method, which was clearly the most precise of all methods, being better than 3.75° for elbow flexion and better than 2.75° for elbow extension across the entire measured ROM range (see Fig 6).

## Discussion

In this study, we investigated the accuracy, precision, and concordance of monocular HPE methods and visual assessments when measuring elbow flexion and extension ROM using a passive-marker OMC system as the reference. This study was conducted on a clinical population with elbow and shoulder pathologies, addressing a gap in the validation of HPE methods, as these methods have rarely been validated in real-world clinical conditions with pathology-affected movement patterns.

### Elbow flexion

All three investigated methods demonstrated a sufficient MDC for clinical measurements of elbow flexion ROM. The MDC of HSMR, RTMW, and SOC all fell clearly below the proposed MCID of 10° for elbow flexion [10], indicating that each of these methods can reliably detect changes of clinical relevance in individual patients within this clinical population.

**Fig 7:**
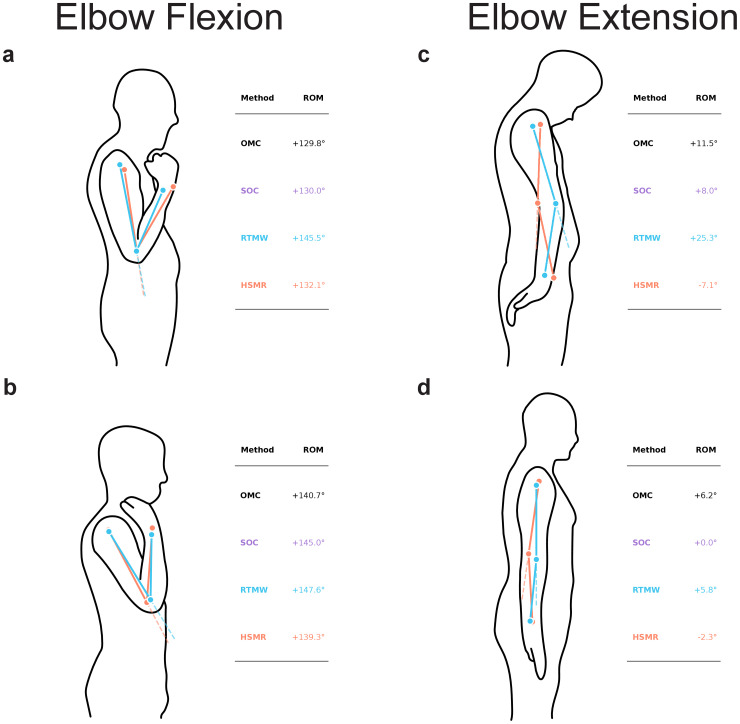
Examples of ROM measurements. Frames from the RGB camera depicting maximal flexion and extension ROM after anonymization. The colored circles indicate the unmodified positions of the joint centers detected by RTMW and HSMR. The values next to each image indicate the measured ROMs for the depicted participant and motion. Note that the angles indicated by the dashed lines correspond to the projection of the elbow angle onto the 2D image plane and that HSMR returned angles from the SKEL biomechanical model, which therefore can differ. a) Large flexion angle with a misleading projection: internal rotation of the upper arm led to the forearm lying outside the image plane and a large overestimation by RTMW. b) Large flexion angle without misleading projection: absence of internal rotation of the upper arm led to the forearm lying entirely within the image plane and thus a smaller overestimation by RTMW. c) Large extension angle with a misleading projection: internal rotation of the upper arm led to the forearm lying outside the image plane and an overestimation by RTMW. In cases of large ROM, HSMR often selected the outermost possible position on the detected human mesh for the wrist and elbow joints. d) Large extension angle without misleading projection: absence of internal rotation of the upper arm led to the forearm lying entirely within the image plane and thus a smaller deviation by RTMW.

The linear mixed-effects models assumed a constant bias across the measurement range, and under this assumption HSMR had the smallest constant bias and the highest CCC of all methods. Its high accuracy metrics, high CCC, and low MDC imply that HSMR was the most accurate and precise method for measuring elbow flexion ROM in this clinical population. The Taffé analysis revealed that HSMR had a decreasing bias for increasing true flexion ROM, switching from overestimation to underestimation at approximately 113° of flexion. SOC approached a bias of zero for increasing flexion angles, and had a smaller absolute value of bias than HSMR for flexion angles above approximately 133°. RTMW clearly showed the largest absolute value of bias of all methods and consistently overestimated flexion angles by approximately 2.5° up to 13°. This behavior could be explained by the fact that RTMW is a 2D HPE method that does not fit a kinematic model to the detected human bodies. Some participants twisted their forearms out of the image plane during the motion by internally rotating their upper arms, which led to the projected elbow flexion ROM appearing larger in the 2D image than it actually was. These misleading projections resulted in an overestimation of flexion angles, in particular those in the upper quartile of measured values (see Fig 7, a). When the forearm remained entirely within the image plane during the motion, the deviation of the projected elbow flexion ROM from the true elbow flexion ROM was smaller even in case of large flexion angles (see Fig 7, b). Cheng et al. mathematically derived the errors when measuring 2D projections of 3D joint angles and found that the error in measuring flexion angles can be substantial when out-of-plane motions are present, which is common in the upper limbs, supporting our findings [42]. In contrast, the SKEL biomechanical model in HSMR led to more accurate flexion angles even in cases of misleading projections of the elbow angle onto the image plane, as it models the entire kinematic chain from the shoulders down to the wrists, leading to the lowest bias among all methods for true flexion angles up to 133° (see Fig 7, a). In cases of large flexion angles and large extension angles, HSMR sometimes tended to predict the wrist joint at nonphysiological positions, seemingly selecting the outermost possible position on the detected human mesh (see Fig 7, a and c), which users requiring accurate joint positions should be aware of. Above flexion angles of around 133°, SOC showed the smallest absolute value of bias of all tested methods, though the difference in absolute values of bias to HSMR was small in this range, just over 2° (see Fig 5).

Surprisingly, RTMW showed the highest precision across almost the entire measured flexion ROM range, which remained nearly constant around 5° to 5.5°. HSMR showed higher precision than SOC for true flexion angles below 129°, and SOC was slightly more precise than HSMR for true flexion angles larger than 129°. It should be noted that the confidence intervals of precision shown by HSMR and SOC overlapped over the entire measured flexion ROM range, which indicates that the difference in precision between these two methods might not be statistically significant.

These results suggest that biomechanical models can substantially increase the accuracy of HPE methods when measuring joint angles in highly flexed positions due to an increased robustness towards these joint angles lying outside of the image plane. Furthermore, the results of the linear mixed-effects models and the Taffé analysis indicate that HSMR is a suitable alternative to visual assessment when measuring elbow flexion ROM, as HSMR had comparable or even better bias and precision over the entire measured flexion ROM range.

### Elbow extension

When measuring elbow extension ROM in this clinical population, none of the investigated methods achieved an MDC below the proposed MCID of 5° for elbow extension [10]. SOC had the lowest MDC of all methods at 5.61° (95% BCa CI: 4.84°–7.53°), with the confidence interval including the proposed MCID of 5°.

Although HSMR displayed the highest accuracy with respect to the mean difference and the median difference, and had the lowest fixed effect magnitude of all methods, HSMR had the highest mean absolute difference, the highest root mean square difference, and the lowest CCC of all methods. These findings indicate a large proportional bias, which was confirmed by the Taffé analysis, showing that HSMR had the greatest proportional bias of all methods. This large proportional bias and the resulting low CCC could be explained by the fact that the SKEL biomechanical model was designed around typical physiological ROM ranges and that large elbow hyperextension, which was present in this patient cohort, lies outside of these design assumptions. Adapting the constraints on the elbow joint in the underlying SKEL model to allow more hyperextension did not have an effect and did not change the extension ROM values determined by HSMR. Notably, some cases of large hyperextension were misidentified as flexion (see Fig 7, c). As previously mentioned, HSMR sometimes predicted the wrist joint at nonphysiological positions for large flexion and extension angles. In hyperextension, this behavior was noticeable for the elbow joint as well, where HSMR seemed to erroneously detect the outermost possible position on the detected human mesh for the wrist and elbow joints (see Fig 7, c). SOC showed the highest CCC and the highest accuracy with respect to the mean absolute difference and root mean square difference. Both SOC and RTMW had similar patterns of decreasing bias for increasing true extension ROM, but SOC showed a smaller absolute value of bias for true extension angles below approximately 7°.

HSMR displayed the lowest precision across nearly the entire measured extension ROM range, which remained almost constant around 8° to 9°. RTMW showed a nearly constant precision of around 6° to 7° across the entire measured extension ROM range, which made it the most precise of the investigated methods for extension angles smaller than −13°. Above true extension angles of −13°, SOC showed the highest precision of the investigated methods.

These results suggest that the current SOC of visual assessment when performed by experienced clinicians can outperform state-of-the-art monocular HPE methods at measuring elbow extension ROM, as the surgeon had a smaller absolute value of bias and higher precision for the large majority of measured extension ROM values. Furthermore, the results indicate that biomechanical models that are designed around typical physiological ROM ranges might not be suitable for measuring extension ROM in clinical populations with elbow hyperextension, and that future improvements to these biomechanical models might try to account for this. RTMW achieved a bias comparable to SOC, with the difference in absolute bias between these two methods being smaller than 2° and a nearly constant precision, which was better than SOC for extension angles smaller than −13° but up to 3° worse than SOC for extension angles larger than −13°. This improved performance of RTMW relative to elbow flexion could be explained by the fact that elbow extension angles typically were smaller in magnitude than flexion angles and therefore less affected when the elbow angle did not lie perfectly in the image plane (see Fig 7, d). When large extension angles were present and participants internally rotated their upper arms, the projected elbow extension ROM could be misleading as well (see Fig 7, c). Therefore, when measuring elbow flexion and extension ROM in the presence of large internal rotation of the upper arm, it is important to be aware of the large projection errors when measuring with monocular HPE methods aimed at the sagittal plane, which could be avoided by re-aiming the camera to be perpendicular to the plane of motion or by using a multi-camera setup. These results suggest that RTMW could be used to measure elbow extension ROM when it is not necessary to achieve clinical accuracy needed for clinical decision-making, such as in sports and fitness tracking, telehealth screening and triage, ergonomic assessment, and group-level research. Future improvements would be necessary to achieve the accuracy and precision of SOC for the entire measurement range and achieve an MDC below the MCID. Such improvements could come from biomechanical models that are compatible with monocular skeleton-based HPE methods, where there appears to be a gap in the current literature. Future biomechanical models should also account for wider joint ranges of motion by adapting their kinematic constraints or including more diverse data in their creation.

### Accuracy of visual assessments

Our investigation found that visual assessment performed considerably better than previously documented. The observed mean absolute differences for elbow flexion ROM of 5.4° and elbow extension ROM of 5.2° (see Table 2) were substantially lower than the 8.4° and 12.2° reported in a comparable study on cadavers [10]. This higher accuracy is likely due to the high experience of the orthopedic surgeon in our study, indicating that the SOC performed in our study likely represents a best-case scenario and once again underscoring the importance of experience on the accuracy of visual estimation.

### Limitations and suggestions

This study has some limitations which should be considered before drawing conclusions. A primary limitation is the use of only one highly experienced orthopedic surgeon for visual assessments. For this reason, our results may not be generalizable to visual assessments by clinicians with other expertise and levels of experience. The primary goal of this study was to validate HPE methods against a reference system and to compare the results with visual assessment. We were not interested in determining the inter-rater and intra-rater reliability of visual assessments, as they have already been reported extensively. For these reasons, we decided to include just one expert rater, representing the best-case scenario to compare with the HPE methods.

Another limitation might be due to the controlled laboratory setting. We avoided having any humans besides the patient being visible in the captured videos and strictly controlled the positions and motions of the patients. Furthermore, the lighting in the laboratory was uniformly bright and the background clutter was minimal. These controlled parameters may not be representative of real clinical settings. Therefore, the accuracy and precision of the investigated HPE methods represent a best-case scenario in terms of factors that influence image quality and may differ from those encountered in real-world settings.

A further limitation comes from the visual appearance of the participants, which could have influenced the results of the HPE methods. We did not control the appearance of the participants except for requiring tight clothing, and we did not recruit participants considering their ethnicity or body metrics such as height, weight, or body mass index. The accuracy and precision of the investigated HPE methods might differ in other study populations. It should be noted once again that this study population included only 10 patients in treatment for elbow pathologies, leading to a skewed distribution of the measured ROM values.

In this study, we only investigated monocular HPE methods due to their high suitability for clinical use, as they have low hardware and setup requirements. This approach was effective because the elbow motions measured in this study could be performed almost entirely within the image plane, thus maintaining accuracy. More complex 3D motions of the shoulder or wrist cannot be easily performed solely within the image plane. In these general 3D cases, it might be necessary to use multi-camera HPE methods to achieve high enough accuracy and precision, which might be investigated in future studies.

It should be noted that seven out of 46 participants were only able to perform two repetitions of the requested three repetitions for both elbow flexion and extension. Both selected statistical methods, i.e., linear mixed-effects models and the Taffé method, can be applied to unbalanced repeated measurements. Due to the small number of missing repetitions, the fact that we did not remove any other repetitions, and the selected statistical methods being able to model such unbalanced data, we do not expect this to have affected the results of this study.

Finally, the passive-marker OMC reference system is not free of measurement error itself, and marker placement variability and soft tissue artifacts are the main sources of errors in such systems [43,44]. The same experienced investigators performed the marker placement in all sessions to reduce its variability. The largest soft tissue artifacts have been reported for regions with large amounts of skin, fat, and muscle, such as the thigh. As the upper extremities tend to have less soft tissue volume and the types of movements performed in this study were slow and controlled, we do not expect a large influence of soft tissue artifacts on the results of this study. Differences in body mass index between participants were not controlled for and might have influenced the results due to an increase in soft tissue artifacts. Nevertheless, we selected passive-marker OMC as the reference system because it is currently considered the gold standard for clinical motion analysis due to its high accuracy compared to other non-invasive methods such as inertial measurement units or markerless systems.

## Conclusion

In this study, we evaluated two different monocular human pose estimation methods using RGB videos and compared their performance against expert visual assessment when measuring elbow flexion and extension ROM. All methods were tested on a clinical population with elbow and shoulder pathologies using passive-marker optical motion capture as the reference system. For elbow flexion, HSMR provided a viable alternative to expert visual assessment in this clinical population, achieving comparable or superior accuracy and precision and a minimal detectable change below the minimal clinically important difference. For elbow extension, both investigated markerless human pose estimation methods could not provide an improvement over expert visual assessment because their accuracy and precision were lower. However, they may still be useful for measurements, which do not require the same level of clinical accuracy. None of the investigated methods, including expert visual assessment, achieved a minimal detectable change below the proposed minimal clinically important difference for elbow extension. We further conclude that biomechanical models can substantially improve the accuracy of monocular human pose estimation, but their joint angle constraints must be well-suited to the task to avoid poor performance.

## Supporting information

S1 Fig Definition of the range of motion used for elbow flexion and extension. (a) The neutral position or zero-degree position in this study corresponded to the forearm being aligned with the upper arm in the direction of gravity when standing upright. This was the reference position for measuring both flexion and extension ROM. In this neutral position, the elbow joint was considered to be at 0° of flexion or 0° of extension, respectively. (b) Regardless of whether a participant previously reached the neutral position, the clinical definition of elbow flexion ROM was the angle starting from the neutral position and ending at the maximal flexion angle. In this case, the clinical definition corresponds to the elbow flexion angle set by the ISB. (c) The clinical definition for elbow extension ROM started from the neutral position and ended at the maximal extension angle. Note that the clinical extension ROM can take negative values when the participant is not able to reach the neutral starting position. The ISB defines elbow (hyper)extension as a rotation of the forearm around the z-axis of the humerus coordinate system by a negative angle. In the case of elbow extension, a decreasing ISB elbow extension angle corresponds to an increasing clinical elbow extension ROM. When starting from the neutral position, converting from the ISB definition of the elbow extension angle to the clinical definition of elbow extension ROM and the reverse can be achieved by changing the sign of the extension angle. The ROM for elbow flexion and extension according to ISB then corresponds to the sum of clinically defined elbow flexion and elbow extension ROM.
(TIF)

S2 Fig Histograms and confidence intervals of accuracy metrics by non-parametric bootstrap simulations. The non-parametric bootstrapping was performed with 10,000 simulations and participant-wise redrawing. Black lines represent the point estimates from a linear mixed-effects model fitted on the original data. The red lines represent the 95% confidence interval using the 2.5% and 97.5% percentile. The green lines represent the bias-corrected and accelerated (BCa) 95% confidence intervals, which are reported.
(TIF)

S3 Fig Diagnostic plots of linear mixed-effects models on original data. Residuals over fitted ROM values, Q-Q plots of the residuals, and Q-Q plots of the random effects of all linear mixed-effects models, which were used to extract the point estimates of bias and accuracy metrics.
(TIF)

S1 Table Accuracy metrics and their 95% BCa confidence intervals. The point estimates of the metrics were computed by fitting linear mixed-effects models on the measured data. The confidence intervals were computed by non-parametric bootstrapping with 10,000 iterations and participant-wise redrawing and BCa correction. Underlined values indicate the best value for each motion.
(PDF)
