# Supplementary figures and images for: Validation of markerless human pose estimation methods for clinical assessment of elbow range of motion
Source: PLoS One. 2026 Jul 23;21(7):e0353801. doi: 10.1371/journal.pone.0353801 (PMC13395372; doi:10.1371/journal.pone.0353801)

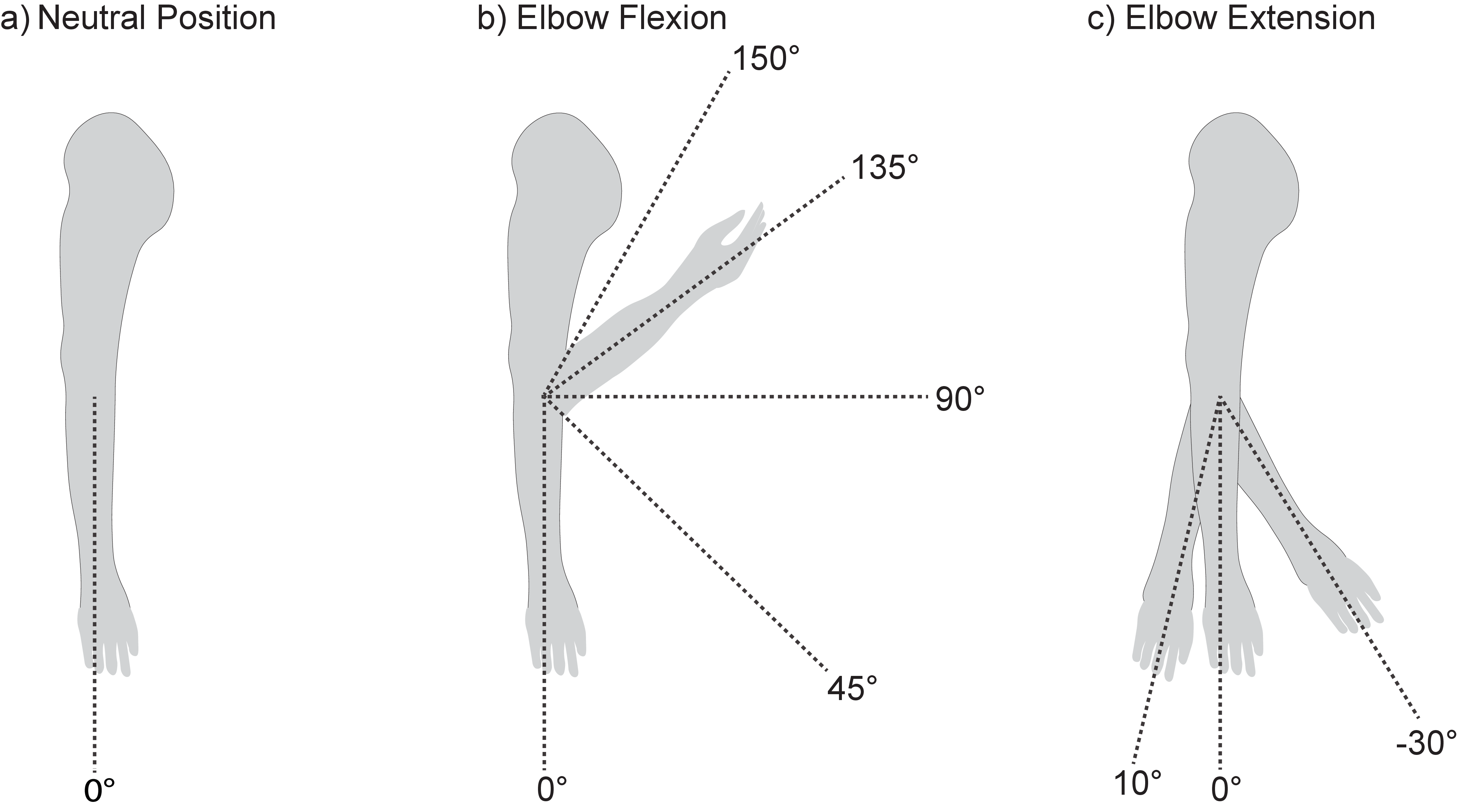

Supplement: S1 Fig — (a) The neutral position or zero-degree position in this study corresponded to the forearm being aligned with the upper arm in the direction of gravity when standing upright. This was the reference position for measuring both flexion and extension ROM. In this neutral position, the elbow joint was considered to be at 0° of flexion or 0° of extension, respectively. (b) Regardless of whether a participant previously reached the neutral position, the clinical definition of elbow flexion ROM was the angle starting from the neutral position and ending at the maximal flexion angle. In this case, the clinical definition corresponds to the elbow flexion angle set by the ISB. (c) The clinical definition for elbow extension ROM started from the neutral position and ended at the maximal extension angle. Note that the clinical extension ROM can take negative values when the participant is not able to reach the neutral starting position. The ISB defines elbow (hyper)extension as a rotation of the forearm around the z-axis of the humerus coordinate system by a negative angle. In the case of elbow extension, a decreasing ISB elbow extension angle corresponds to an increasing clinical elbow extension ROM. When starting from the neutral position, converting from the ISB definition of the elbow extension angle to the clinical definition of elbow extension ROM and the reverse can be achieved by changing the sign of the extension angle. The ROM for elbow flexion and extension according to ISB then corresponds to the sum of clinically defined elbow flexion and elbow extension ROM. (TIF) [file pone.0353801.s001.tif]

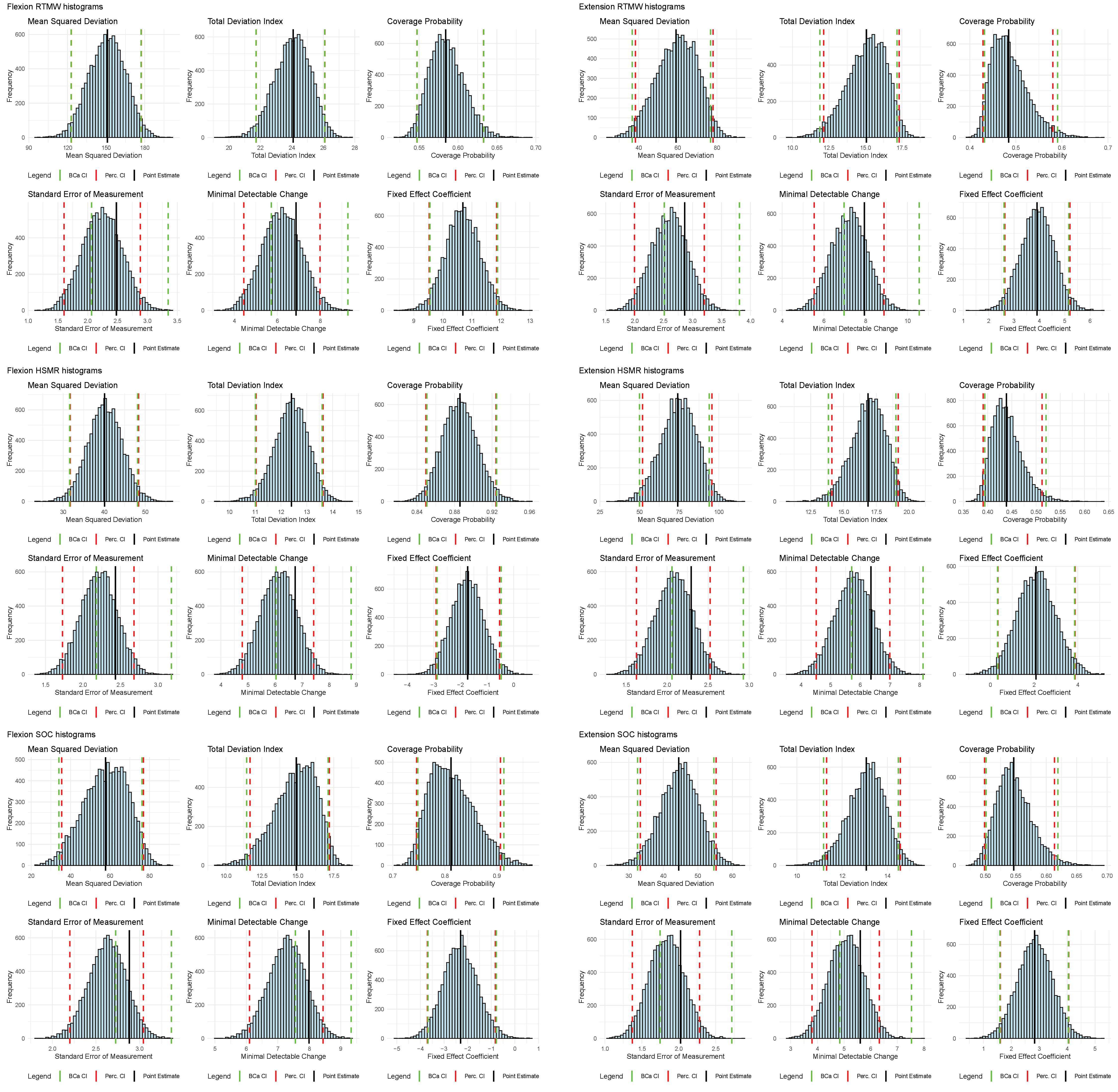

Supplement: S2 Fig — The non-parametric bootstrapping was performed with 10,000 simulations and participant-wise redrawing. Black lines represent the point estimates from a linear mixed-effects model fitted on the original data. The red lines represent the 95% confidence interval using the 2.5% and 97.5% percentile. The green lines represent the bias-corrected and accelerated (BCa) 95% confidence intervals, which are reported. (TIF) [file pone.0353801.s002.tif]

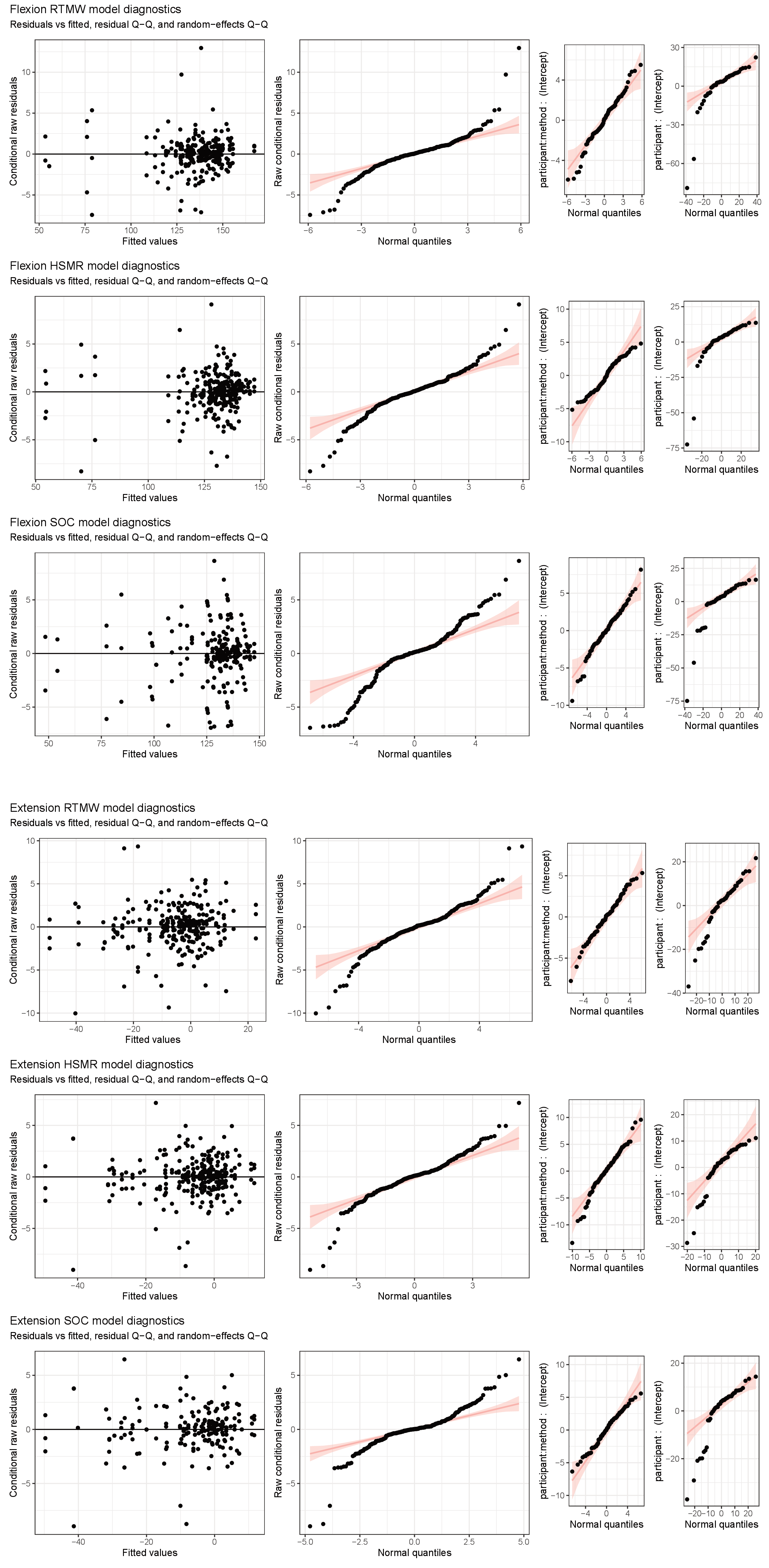

Supplement: S3 Fig — Residuals over fitted ROM values, Q-Q plots of the residuals, and Q-Q plots of the random effects of all linear mixed-effects models, which were used to extract the point estimates of bias and accuracy metrics. (TIF) [file pone.0353801.s003.tif]
