# Supplementary material for: Validation of markerless human pose estimation methods for clinical assessment of elbow range of motion
Source: PLoS One. 2026 Jul 23;21(7):e0353801. doi: 10.1371/journal.pone.0353801 (PMC13395372; doi:10.1371/journal.pone.0353801)
Supplement: S1 Table — The point estimates of the metrics were computed by fitting linear mixed-effects models on the measured data. The confidence intervals were computed by non-parametric bootstrapping with 10,000 iterations and participant-wise redrawing and BCa correction. Underlined values indicate the best value for each motion. (PDF) [file pone.0353801.s004.pdf]

S4 Table. Accuracy metrics and their 95% BCa confidence intervals. The point estimates of the metrics were computed by fitting linear mixed-effects models on the measured data. The confidence intervals were computed by non-parametric bootstrapping with 10,000 iterations and participant-wise redrawing and BCa correction. Underlined values indicate the best value for each motion.

		RTMW	HSMR	SOC
Elbow flexion ROM	CCC	0.80 (0.44,0.91)	<u>0.92</u> (0.66,0.97)	0.91 (0.75,0.96)
	MSD ($°^2$)	151.01 (122.85,177.18)	<u>40.08</u> (31.57,48.13)	57.74 (34.05,76.22)
	TDI (°)	24.09 (21.72,26.09)	<u>12.41</u> (11.01,13.60)	14.89 (11.44,17.11)
	CP (%)	58.42 (54.75,63.30)	<u>88.58</u> (85.05,92.49)	81.18 (74.80,91.33)
	SEM (°)	2.48 (2.06,3.35)	<u>2.43</u> (2.18,3.18)	2.88 (2.73,3.37)
	MDC (°)	6.87 (5.72,9.28)	<u>6.74</u> (6.04,8.81)	7.99 (7.56,9.33)
Elbow extension ROM	CCC	0.83 (0.74,0.90)	0.72 (0.57,0.80)	<u>0.86</u> (0.76,0.91)
	MSD ($°^2$)	59.12 (36.74,76.74)	74.08 (50.00,93.94)	<u>44.45</u> (32.54,54.62)
	TDI (°)	15.07 (11.88,17.17)	16.87 (13.86,19.00)	<u>13.07</u> (11.18,14.49)
	CP (%)	48.45 (43.19,59.07)	43.87 (39.41,52.06)	<u>54.67</u> (50.13,61.92)
	SEM (°)	2.86 (2.51,3.81)	2.29 (2.06,2.93)	<u>2.02</u> (1.75,2.72)
	MDC (°)	7.93 (6.96,10.56)	6.36 (5.71,8.12)	<u>5.61</u> (4.84,7.53)
